# Bioinductive collagen implants facilitate tendon regeneration in rotator cuff tears

**DOI:** 10.1186/s40634-022-00495-7

**Published:** 2022-06-08

**Authors:** Jorge Alberto Camacho-Chacon, Jorge Cuenca-Espierrez, Victor Roda-Rojo, Adrian Martin-Martinez, Jose Manuel Calderon-Meza, Ramiro Alvarez-Alegret, Carlos Martin-Hernandez

**Affiliations:** 1 Instituto Aragones de ortopedia, traumatología y medicina regenerativa (IATR), C/ Los Andes 1 Bajo 2, 50012 Zaragoza, Spain; 2 Hospital Universitario Miguel Servet, IIS Aragon, Paseo Isabel la Católica 1-3, 50009 Zaragoza, Spain

## Abstract

**Purpose:**

To evaluate the clinical outcomes, MRI imaging and histological characteristics of biopsy samples of the tendon from patients in whom rotator cuff repair was previously performed with a bioinductive type I bovine collagen implants.

**Methods:**

Prospective study of 30 patients with partial or complete rotator cuff tears who underwent arthroscopic repair and augmentation with a resorbable type I bovine collagen implant. Preoperatively and at 6 and 12 months after surgery, the VAS, ASES and Constant-Murley scores were evaluated and an MRI study was performed. At 6 months, biopsies of the resulting tissue were obtained and examined histologically.

**Results:**

Patients experienced statistically significant and sustained improvement from baseline for all scores and the mean tendon thickness increased by 1.84 mm. Magnetic resonance imaging evidence of complete healing was found in 27 patients and a considerable reduction in defect size, greater than 50%, was shown in 3. In all samples obtained, the new tissue generated had the histological appearance of a tendon, and was indistinguishable from the native tendon. There was no evidence of any remaining collagen implant.

**Conclusions:**

Biopsies of tissue formed from bioinductive type I bovine collagen implants showed, six months after surgery, the generation of a neotendon indistinguishable from the native one. Histology and MRI imaging, revealed complete integration of the implant and absence of inflammatory or foreign body reactions. The clinical parameters, thickness and MRI signal of the tendon improved significantly at 6 months, regardless of the type and size of the tear, and remained unchanged until 12 months.

**Level of evidence:**

Level IV, case series.

## Introduction

Rotator cuff injuries are the most common shoulder pathology and can be found in 30% to 50% of the population over 50 years of age [16]. They often require surgical treatment to recover function and reduce pain. Symptomatic partial and full-thickness tears, with poor-quality tissue, are often a challenge for orthopaedic surgeons, and there is currently no consensus on a single protocol for their management [14, 18]. Despite advances in repair techniques and fixation devices, re-rupture rates remain high due to several factors, such as age, degenerative changes, the size of the tear and the quality of the tendon tissue [12]. Surgical repair can be difficult or even impossible in the presence of massive tears. In an attempt to improve the strength of surgical repair, new materials and surgical techniques have been proposed, aiming to reproduce the anatomical footprint of the cuff. Despite these technical advances, several studies have shown the persistence of a high repair failure rate of up to 45% [11].

In recent years, the focus of research has changed from the mechanical improvement of suture techniques to ways of improving the biological environment in the repair area to recreate the normal footprint of the tendon and to decrease the tension of the tissue under repair. This change can lead to a better prognosis after the repair of the rotator cuff tendon and to a reduction in the incidence of new tears [13]. Among other methods, patch augmentation techniques have been shown to improve vascularization and collagen formation at the site of tendon repair [2, 7, 17, 24]. A highly organized, highly porous, reconstituted type I bovine collagen bioinductive implant has been designed to promote the regeneration of new host tissue. This implant is placed on the bursal surface of partial [6] or full thickness [4] cuff lesions. It is not structural and does not provide any tensile strength, but improves the formation of collagen at the repair site, thus decreasing tension in the repaired tendon. Experimental studies in animals have documented the evolution and biocompatibility of the implant as well as the maturation and orientation of the newly induced tissues over time [23, 25] and imaging studies with ultrasound [24] and MRI [5, 21, 22] have shown the generation of new tissue that matured and became radiologically indistinguishable from the underlying tendon, with complete healing or at least a considerable reduction in the size of the defect. A single sequential histological study of biopsies of collagen implants extracted from human subjects revealed cellular incorporation, tissue formation and maturation, implant resorption and, finally, the generation of tissue with a histological tendon appearance [1].

The clinical results at one and two years have shown a significant improvement in all scales evaluated [5, 6, 15, 21, 22], with comparable implant efficacy regardless of the size of the underlying tear.

The purpose of this study is to evaluate the clinical outcomes, MRI imaging and histological characteristics of biopsy samples of the tendon from patients in whom rotator cuff repair was previously performed with a bioinductive type I bovine collagen implants. Previous studies have focused on clinical and imaging results. We believe that further studies showing the correlation between these and histologic characteristics of the newly formed tissue were necessary.

The hypothesis was that the resulting tissue would show histological and imaging characteristics similar to those of a normal tendon, with a significant improvement in the clinical parameters evaluated.

## Materials and methods

A prospective study of 30 consecutive patients with chronic degenerative rotator cuff tears was conducted.

Patients with partial-thickness and complete tears of the supraspinatus tendon were recruited. The sample consisted of 16 women (46.7%) and 14 men (53.3%), aged between 35 and 74 years (mean 56.5 years). 22 shoulders (70%) were right and 8 (30%) left.

The inclusion criteria were: Patients older than 18 years with the diagnosis of partial or total rupture of the rotator cuff with failure of conservative treatment (analgesics, anti-inflammatory medication, and physical therapy) after 6 months, absence of previous surgeries, consent for surgical intervention and specific for surgery and the performance of percutaneous biopsy 6 months after surgery and absence of infectious complications after arthroscopy.

Exclusion criteria included known hypersensitivity to bovine collagen, recent steroid use, insulin-dependent diabetes, heavy smoking, genetic collagen disease, chronic inflammatory disease and previous cuff surgery. Patients with shoulder instability, grade 3 or greater chondromalacia, were also excluded.

All of them underwent surgery performed by the same surgeon through arthroscopic repair, when required, and augmentation with a bioinductive bovine type I collagen implant (Regeneten®, Smith & Nephew, Andover MA). Informed consent was obtained from each patient in the study.

Patients were evaluated clinically and by means of a preoperative MRI study at 6 months and at final follow-up, 12 months after the intervention. Six months after surgery, a percutaneous ultrasound-guided biopsy of the repair tissue was performed under local anaesthesia.

### Clinical evaluation

Clinical evaluation was performed preoperatively and at 6 and 12 months after surgery using the pain visual analogue scale and the Standardized American Shoulder and Elbow Surgeons (ASES) and Constant-Murley scores.

### Surgical technique

With the patient placed in the lateral decubitus position and under general anaesthesia, following arthroscopic assessment of the glenohumeral joint, a subacromial bursectomy was performed. Intraoperative arthroscopic evaluation of the rotator cuff pathology and visual confirmation of the tear size were done. Both were recorded by the surgeon to confirm the eligibility of each patient. Tears were graded according to Ellman arthroscopic classification [8]. For full-thickness tears, Bateman classification was used [3].

In case of cuff impingement, acromioplasty (7 patients) was performed prior to placement of the implant. Biceps tenotomy were done in case of degeneration, partial biceps tendon tears, atrophy or subluxation (27 patients).

Partial cuff tears were treated with debridement only. In case of complete tears, double-row transosseous equivalent rotator cuff repairs were performed.

The entire implant was arthroscopically delivered through the lateral portals on the bursal surface of the supraspinatus tendon using custom designed instrumentation according to a previously described technique [4, 20]. Depending on the size of the tear, the surgeon selected a collagen implant sized either 20 × 24 mm (“medium”) or 25 × 30 mm (“large”) to cover the tear and the width of the supraspinatus tendon. Employing proprietary, single-use disposable instruments, the surgeon secured the implant to the tendon with polylactic acid anchors and the implant to the greater tuberosity with polyether ether ketone (peek) bone anchors completely covering the tear.

### Rehabilitation

The postoperative rehabilitation protocol depended on the type of tear.

In patients with partial tears, the immobilization of the shoulder was removed as soon as possible, and they were allowed to progress in the movement according to tolerance, progressing from passive to active-assisted movement and to active movement during the first 4 weeks. After these, no restrictions on movement or use of the arm were imposed.

Patients with complete tears remained with the shoulder immobilized in a sling for 4 weeks while performing elbow flexion and pendulum movements starting at 2 weeks. Beginning at 4 weeks, self-assisted passive mobilization and exercises with internal rotation to the abdomen and external rotation to the neutral position were initiated. After 6 weeks, no restrictions on movement or use of the arm were imposed.

### MRI evaluation

The magnetic resonance images were obtained on the same Siemens Magnetom Vida (Siemens Healthineers AG. Erlangen, Germany) 3 Tesla scanner, preoperatively and at 6 and 12 months, without contrast, with the shoulder in slight external rotation using a field of view of 12 cm, a thickness of the slices of 2 mm and a gap between cuts of 0.2 mm. The images included coronal, sagittal and axial sections with proton density (PD), and with T2 weighted scans, both with and without fat saturation. All magnetic resonance images were evaluated blindly by a single musculoskeletal radiologist.

The size of the tears and muscle fatty infiltration, according to Goutallier classification [9], were recorded. The measurements of the tendon thickness were performed in partial tears in the area of the tear and in the area adjacent to the joint margin of the supraspinatus tendon according to the method described by Bokor et al. [5]. In complete tears, measurement of the size of the defect was performed by recording tear width and retraction measured in millimetres. Width was defined as the largest distance between tear margins in the anterior-posterior (AP) direction on an oblique sagittal view, and retraction was defined as the largest distance between the lateral portion of the greater tubercle of the humerus and the free margin of the rotator cuff on an oblique coronal view. All postoperative measurements were performed as close as possible to the original tear in which the preoperative measurements were taken.

The evaluation of the filling of the defects was qualitatively performed (absent, partial, total) to determine if the tears progressed, remained or their size was reduced.

### Histological study

At 6 months after the intervention, after clinical re-evaluation and performance of MRI, 29 patients underwent percutaneous biopsy with a 1.5 mm biopsy punch under local anaesthesia. The donor site was located under ultrasound control by selecting the intermediate portion of the neotendon located between the two bone anchors anchored in the anterior and posterior middle area of the implant. A patient with a complete tear underwent reoperation for arthrofibrosis after 6 months to lyse the adhesions, and a new arthroscopy was performed in which the newly formed tissue from the implant could be visualized and a sample of it obtained.

The specimens resulting from the biopsies had an average size of 3.5 mm^2^ and were fixed in 4% buffered formaldehyde for 24 h and processed in paraffin with the ordinary 16-h protocol (dehydration in increasing concentrations of alcohols, clearing in xylene, and paraffin-embedding). The histological sections, 3 μm thick, were stained with the routine hematoxylin-eosin technique and were evaluated under an optical microscope blindly by two independent pathologists.

The following parameters were evaluated: tissue type, inflammation, fibrosis, presence of foreign bodies (including collagen implants), and ischemia.

### IRB approval

This study was approved by the Institutional Review Board with number 07/2021.

### Statistics

Descriptive statistics were used to summarize patient demographics, intraoperative surgical evaluations, and clinical outcomes. Changes between initial and postoperative clinical results (VAS, ASES and Constant-Murley) and tendon thickness were compared using the nonparametric Mann–Whitney-Wilcoxon test. All statistical calculations were performed with IBM® SPSS® Statistics version 19. All *P* values were considered significant at a level of significance of 0.05.

## Results

Of the 30 patients included, 12 showed complete tears that were treated by bursectomy, double row repair with transosseous-equivalent technique and biological augmentation with the implant. Eighteen patients had partial ruptures of the cuff (4 articular, 4 bursal and 10 intrasubstance) and were treated by bursectomy, debridement and augmentation.

There were no significant differences in age between men and women (*P* = 0.089). The most frequent rupture in men was complete, while in women, it was intrasubstance.

None of the patients reported problems after the biopsy.

### Clinical results

Shoulder pain and function improved significantly during the postoperative year. The VAS score improved significantly (*P* = 0.003), from 7.23 ± 0.77 at the beginning to 0.57 ± 1.13 at six months and 0.27 ± 0.94 at one year, and the ASES and Constant scores also improved significantly from 48.03 ± 1.18 to 85.93 ± 7.25 at six months and 87.80 ± 7.00 at one year (*P* = 0.001) and from 58.60 ± 1.61 to 85.37 ± 6.51 at six months and 90.23 ± 5.88 at one year (*P* = 0.001), respectively.

No statistical differences were found between complete and partial tears.

### MRI results

Image characteristics of the tears are summarized in Tables 1 (partial) and 2 (full-thickness).

**Table 1:** Characteristics of the partial-thickness tears according to Ellman and Goutallier classifications

Location	Grade	Goutallier
Articular Sided	III	1
Articular Sided	II	1
Articular Sided	III	1
Articular Sided	II	2
Bursal Sided	III	1
Bursal Sided	III	2
Bursal Sided	II	2
Bursal Sided	III	1
Intrasubstance	III	1
Intrasubstance	III	2
Intrasubstance	II	2
Intrasubstance	II	2
Intrasubstance	III	1
Intrasubstance	III	2
Intrasubstance	II	1
Intrasubstance	III	2
Intrasubstance	II	1
Intrasubstance	III	2

**Table 2:** Characteristics of the full-thickness tears according to Bateman and Goutallier classifications

Description	Grade	Goutallier
Small	I	1
Massive	IV	3
Large	III	2
Large	III	2
Large	III	2
Large	III	2
Large	III	2
Large	III	2
Massive	IV	2
Massive	IV	2
Massive	IV	2
Large	III	1

Preoperatively, in full-thickness tears, the average retraction was 17.01 ± 6.13 mm and the average width measured was 13.59 ± 3.10 mm.

**Fig. 1:**
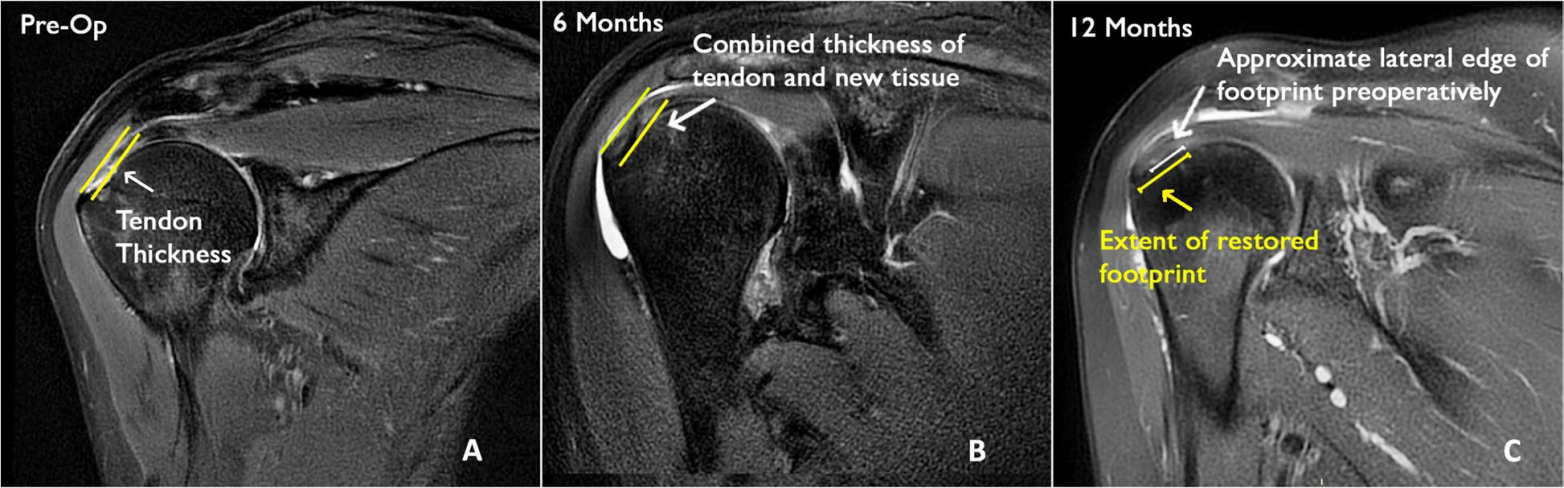
Coronal T2 weighted MRI image of a partial articular tear (**A**) and results after 6 (**B**) and 12 months (**C**), demonstrating the location (arrows) of the measurement of the combined thickness of the layer of new tissue and the underlying tendon

At six months after surgery, there was a significant increase (*P* = 0.001) in the induction of new tissue of the rotator cuff, going from a mean preoperative thickness in partial tears of 4.18 ± 0.29 mm to 6.02 ± 0.29 mm with an average increase in tendon thickness of 1.84 ± 0.29 mm. The MRI signal of the neotendon was indistinguishable from the underlying tendon at 6 months and kept unmodified at 12 months (Fig. 1).

In all patients, there was filling of the defects at 6 months; 27 of them showed complete filling, and 3 of them had partial joint tears and partial filling greater than 50%.

None of the patients showed evidence of propagation of tendon rupture or degeneration or variations in thickness at 12 months.

### Histological results

**Fig. 2:**
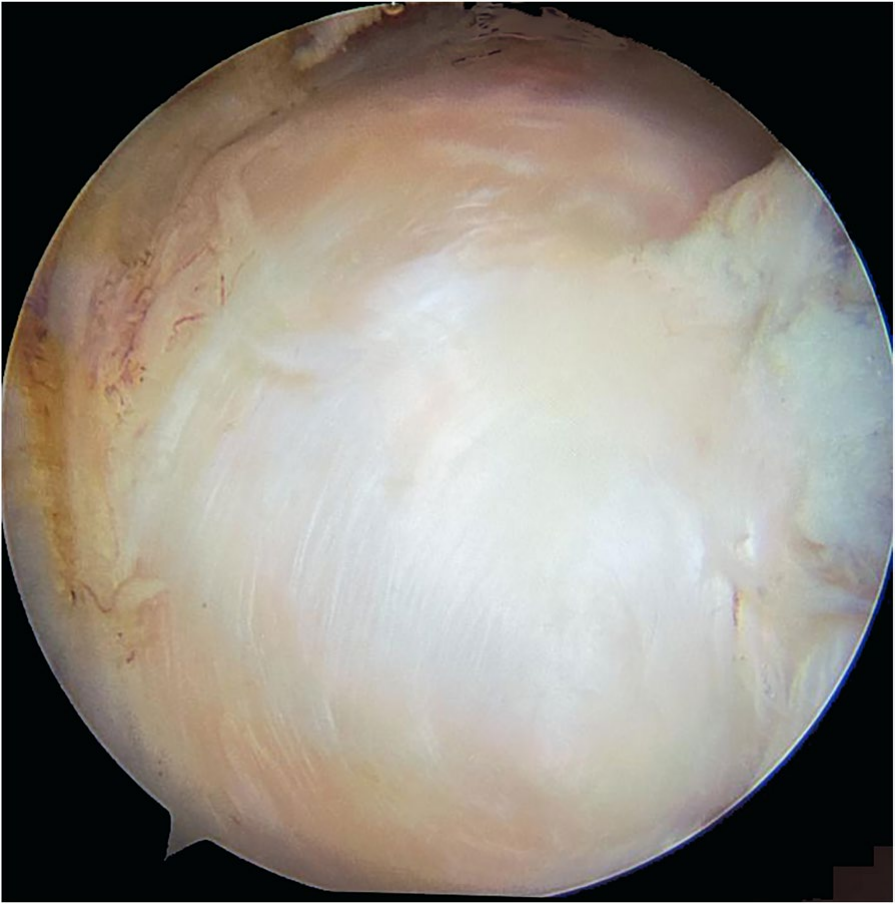
Macroscopic appearance of the repair of a complete tear at 6 months follow-up shows complete healing of the lesion. The second look arthroscopy was performed in the patient who was reoperated on due to arthrofibrosis

The macroscopic examination of the repair site in the patient who underwent reoperation at six months revealed complete healing of the lesion with a layer of new tissue on the surface of the supraspinatus tendon and its humeral footprint. The new tissue adhered to the rest of the cuff and presented an appearance indistinguishable from a healthy tendon (Fig. 2).

**Fig. 3:**
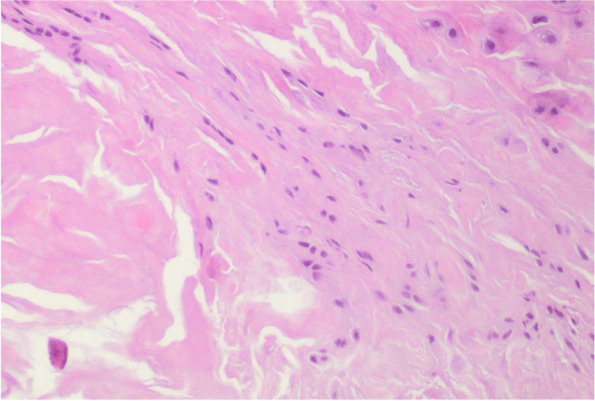
Light photomicrograph of the newly regenerated host tissue by the implant at 6 months. This is dense, regularly oriented connective tissue. There was no evidence of any remnants of the collagen implant. (Hematoxylin and eosin) × 100. Pathologists reported it as “Ligamentous-Tendinous fibrous tissue without relevant morphological lesions”

At 6 months, the newly generated tissue had the histological appearance of a tendon in all samples obtained, mature fibrous connective tissue with parallel rows of fibroblasts within parallel bundles of collagen fibres indistinguishable from the native tendon (Fig. 3). The pathologists confirmed the absence of inflammatory, scarring or ischemic changes in all the specimens, and there was no evidence of foreign body reaction in the tissue samples. There was no evidence of any remains of the collagen implant.

## Discussion

The importance of this study is that clinical and imaging results have been completed with the histological analysis of the repair tissue, showing an integral evolution to the healing of the lesion.

The results of this study show the ability of the bioabsorbable collagen implant to repair both partial and full thickness tears of the rotator cuff. Healing of these defects is associated with the induction of a new tissue histologically indistinguishable from the native tendon after the arthroscopic placement of an implant on the bursal surface of the injured cuff tendon, with a significant clinical improvement and an MRI image of defect filling and increased thickness of the surrounding tendon, corroborating the proposed hypothesis.

Several studies have studied the evolution of bovine collagen implants in animals as in humans, observing various stages in their maturation. In a preclinical study in sheep performed on partial tears [25], after 6 weeks of implantation, the gaps in the collagen scaffold were completely filled with fibrovascular tissue. The fibres of the collagen implant were clearly evident and distinguished from the new tissue growth, showing no evidence of inflammatory reaction or foreign body. At 12 weeks, a layer of new connective tissue covered the upper surface of the tendon, and the implant showed partial resorption, with its fibres being occasionally visible. Histologically, this new tissue was composed of fibroblasts and regularly oriented collagen fibres. After 26 weeks, the layer of new connective tissue was mature and regularly oriented and of a similar thickness to the surrounding tendon, and the fibres of the implant had completely disappeared.

The only histological study performed in humans [1] reflected exactly the same tissue evolution in the same time periods, showing at 6 months the presence of dense and regularly oriented connective tissue that contained parallel rows of fibroblasts within parallel bundles of collagen fibres, the histological definition of a tendon that did not show any presence of residual implant material. Other authors, using ultrasound and MRI [24], also defined this time period as that in which the implant had been completely integrated into the native tissue. For our prospective study, we decided that the appropriate time to perform the biopsy would be at 6 months, at which time we could evaluate both the maturation of the repair tissue and its possible degeneration. On the other hand, for clinical analysis and MRI, a follow-up period of 1 year is the appropriate standard of care in clinical practice to determine our primary variables because there are currently no clinically significant improvements in the patients undergoing a rotator cuff repair [26].

The fact that the bioimplant has been shown to be effective both in partial tears [5, 6, 15] and in total and massive tears [4, 15, 24] allowed us to select a sample including all types of tears, which finally showed no differences between them in the histological characteristics of the repair tissue or in the clinical evolution or MRI imaging of the patients.

The clinical results of this study are comparable to those already published [4–6, 15, 19, 21, 22, 24] and have shown a significant improvement in pain and function of the shoulder, which stabilizes after six months and is maintained after one year. The MRI images also showed a regenerative capacity of the implant facilitating the restoration of the normal footprint of the tendon in the humerus and maintaining the integrity of the repair for 12 months in all patients.

Biopsies of the newly formed tissue from the implant allowed histological confirmation of its biocompatibility. None of the patients included in the study, with the exception of the reported arthrofibrosis, manifested negative effects of either the index procedure or the biopsy performed. The safety of xenografts has been questioned since the study by Iannotti et al. [10] in which a porcine product used to complement rotator cuff repair showed a severe tissue reaction and rejection, but the bioinductive implant used in our study was purified bovine collagen and, as in this study, and the rest of the studies performed with this implant, no rejection or foreign body reactions were found.

Of the complications described in the literature (capsulitis [4], adhesive capsulitis [5], biceps tendinitis [5], bursitis [5], superficial infection [5], scapular dyskinesis [24], infection [15] and intra-articular loose body [15]), only adhesive capsulitis appeared in our series.

Our rate of re-ruptures has been zero, lower than that of other published series [15], but it is necessary to consider that the size of our sample is much smaller.

The follow-up is short, so we cannot rule out long-term tissue degeneration, and a longer follow-up would help to evaluate the viability of the repair tissue, although published studies using imaging techniques agree that its characteristics and improved function persist unchanged two years after surgery [5, 21, 22, 24].

### Limitations

This study has several limitations, especially a short follow-up, a small sample size and the lack of a control group. The patients were not randomized, nor was their treatment blind.

A possible limitation, the location of the biopsy areas, was avoided by using ultrasound to determine the donor site. The ultrasound visibility of the PEEK staples used for implant fixation offered us great security for the selection of the extraction area.

## Conclusions

Biopsies of tissue formed from bioinductive type I bovine collagen implants showed, six months after surgery, the generation of a neotendon indistinguishable from the native one. Histology and MRI imaging, revealed complete integration of the implant and absence of inflammatory or foreign body reactions.

The clinical parameters, thickness and MRI signal of the tendon improved significantly at 6 months, regardless of the type and size of the tear, and remained unchanged until 12 months.
